# Selection and Validation of Reference Genes for qRT-PCR in *Lentinula edodes* under Different Experimental Conditions

**DOI:** 10.3390/genes10090647

**Published:** 2019-08-27

**Authors:** Yi Luo, Gangzheng Wang, Chen Wang, Yuhua Gong, Yinbing Bian, Yan Zhou

**Affiliations:** 1 Institute of Applied Mycology, College of Plant Science and Technology, Huazhong Agricultural University, Wuhan, Hubei 430070, China; 2 State Key Laboratory of Applied Microbiology Southern China, Guangdong Provincial Key Laboratory of Microbial Culture Collection and Application, Guangdong Open Laboratory of Applied Microbiology, Guangdong Institute of Microbiology, Guangzhou 510070, China

**Keywords:** *Lentinula edodes*, reference gene, abiotic stress, biotic stress, growth and development

## Abstract

*Lentinula edodes* is the most consumed mushroom in Asia due to its nutritional and medicinal values, and the optimal reference gene is crucial for normalization of its gene expression analysis. Here, the expression stability of 18 candidate reference genes (CRGs) in *L. edodes* was analyzed by three statistical algorithms (geNorm, NormFinder and BestKeeper) under different stresses (heat, cadmium excess and *Trichoderma atroviride* infection), different substrates (straw, sawdust and corn stalk) and different development stages (mycelia, primordia and fruit bodies). Among the 18 CRGs, *28S*, *Actin* and *α-tub* exhibited the highest expression stability in *L. edodes* under all conditions, while *GPD*, *SPRYP* and *MSF* showed the least stable expression. The best reference gene in different conditions was different. The pairwise variation values showed that two genes would be sufficient for accurate normalization under different conditions of *L. edodes*. This study will contribute to more accurate estimation of the gene relative expression levels under different conditions using the optimal reference gene in qRT-PCR (quantitative reverse transcription polymerase chain reaction) analysis.

## 1. Introduction

With the advantages of high sensitivity and repeatability as well as dynamic quantification range, quantitative reverse transcription polymerase chain reaction (qRT-PCR) is widely used to analyze and validate the expression of target genes [1,2,3]. Nevertheless, the results of qRT-PCR were usually not accurate because of errors caused by many factors such as the efficiency of complementary DNA (cDNA) synthesis and amplification, gene expression normalization, and so on [4,5]. In order to minimize those errors, one or more genes which are usually stably expressed in any experimental conditions are applied in qRT-PCR analysis [5,6]. However, the increasing evidence demonstrated that the expression levels of many tested internal genes in plants and animals as well as fungi were relatively constant only in specific cells or experimental conditions [7,8,9]. The application of unstable internal reference genes will result in significant variations or differences in the qRT-PCR results [10]. Hence, it is necessary to identify and analyze the stability of the reference genes under different conditions to guarantee the accuracy and reliability of qRT-PCR results.

For hundreds of years, *Lentinula edodes* has been used as decoctions and essences or as alternative medicine in many Asian countries such as China, Korea, Japan, etc. [11]. In addition, *L. edode* is the most widely cultivated edible fungi in the world because of its nutritional properties and effective bioconversion of agricultural wastes [12,13]. As a white-rot fungus, the genes in *L. edodes* were identified to encode high-efficiency extracellular enzymes, including laccases and peroxidases as well as lignocellulose enzymes [14,15,16], suggesting the potential application of *L. edodes* in many bioremediation processes in the future. Omics analysis and genetic transformation have obtained some significant achievements in *L. edodes* [17,18,19,20,21]. These results can be used to select and validate the suitable reference genes for further analysis of qRT-PCR results in metabolite biosynthesis and signal transduction pathways. As a matter of fact, qRT-PCR has been widely used to dissect the expression levels of mushroom genes under different conditions [22,23,24,25]. In terms of *L. edodes*, *β-tub* was the most stable reference gene for the mycelia under 37 °C high-temperature stress [26], and *18S* and *Rpl4* were the most stable reference genes for different development stages and under various nutrient conditions, respectively [27]. Nevertheless, the candidate reference genes with relatively constant expression levels under biotic and abiotic stresses and different substrates have not been reported systematically in *L. edodes*.

Heat stress dramatically affects the protein stability, cytoskeleton structure, metabolite biosynthesis, cell membrane and cell integrity in organisms [28,29]. At an ambient temperature higher than 23 °C, pileus size and production of *Agaricus bisporus* fruiting bodies were dramatically decreased [30]. In the presence of high temperatures, the mycelial viability of *L. edodes* becomes weak, leading to areas of rotted logs or bags induced by pathogens like *Trichoderma* sp. and a big loss in production and quality [31,32]. *L. edodes* has higher capacity than plants and animal-derived food in absorbing Cd ion, and the mechanisms for how to remove Cd ion have been reported in several studies [33,34]. However, the molecular mechanism for the response of *L. edodes* to abiotic and biotic stresses as well as different substrates remains unclear. In order to understand this mechanism, it is necessary to select and validate the most suitable reference genes for gene expression analysis under different biotic and abiotic stresses as well as substrates. 

In this study, eighteen candidate reference genes (CRGs) were systematically selected from previous studies and *L. edodes* transcriptome data (unpublished data). The expression stabilities of candidate reference genes were analyzed by geNorm, NormFinder and BestKeeper under 40 °C heat stress, 5 mg/mL Cd(NO_3_)_2_, *T. atroviride* infection, different development stages and substrates [35]. This work aims to identify the most suitable reference genes for future gene expression analysis under different biotic/abiotic stresses, substrates and development stages.

## 2. Materials and Methods

### 2.1. Strain, Culture Conditions and Sample Collection

In treatment with different carbon sources, *L. edodes* strain W1 (ACCC50926) was cultured separately at 25 °C on sawdust medium [21], straw medium (33.3 g straw, 20 g wheat bran, 2 g gypsum and 20 g agar in 1 L sterilized water) and corn stalk medium (33.3 g corn stalk, 20 g wheat bran, 2 g gypsum and 20 g agar in 1 L sterilized water). For abiotic and biotic stress treatment group, mycelia of *L. edodes* strain W1 cultured on sawdust medium were treated under 40 °C heat stress for 24 h, and mycelia growing on the PDA (potato dextrose agar) medium were subjected to *Trichoderma atroviride* infection for 24h. Meanwhile, mycelia cultured on PDA medium 5 mg/mL Cd(NO_3_)_2_ were also collected. Meanwhile, primordia and fruiting bodies of *L. edodes* strain W1 were also collected according to Xiang’s report [27]. After collection, all samples were immediately frozen in liquid nitrogen and stored at −80 °C for RNA extraction.

### 2.2. RNA Isolation and cDNA Synthesis

Total RNA was extracted from all samples using the RNAiso Plus Kit (TaKaRa, Dalian, China) according to the manufacturer’s instruction. The RNA integrity and quantity were checked by 1% agarose gel electrophoresis and the NanoDrop DS-11 Spectrophotometer (Applied Denovix, Madison, WI, USA). The RNA samples with the OD_260_/OD_280_ value between 1.8 and 2.2 were reverse transcribed into cDNA using HiScript^®^ II Q RT SuperMix for qPCR (+gDNA wiper) (Vazyme Biotech, Nanjing, China) according to the instructions of the manufacturer.

### 2.3. Selection of Candidate Reference Genes and Primer Design

Based on previous studies [26,27], sequences of eighteen genes, including seven house-keeping genes and eleven novel candidate genes, were downloaded from the genome database of *L. edodes* [16] and used for primer design. The detailed information of these genes was displayed in Table 1. Primers were designed using Primer Premier 5.0, and the length of PCR amplification products varied from 100 to 253 base pairs (Table 1).

### 2.4. qRT-PCR, Amplification Efficiency and Data Analysis

The qRT-PCR reaction was performed using CFX Connect Real-Time system (Bio-Rad, Hercules, California, USA). The PCR amplification mixture contained 1 μL of cDNA, 5 μL of AceQ^®^ qPCR SYBR^®^ Green Master Mix (Vazyme Biotech, Nanjing, China), 2.5 μL of 10 nM primer mixture and 1.5 μL ddH_2_O. The PCR reaction was performed with an initial denaturation for 5 min at 95 °C; 40 cycles of 30 s at 95 °C, annealing at 60 °C for 30 s; an extension at 72 °C for 20 s. The melting curve (60–95 °C) was used to determine the specificity of every qRT-PCR reaction. All qRT-PCR reactions were performed in biological and technical triplicates. The standard curves were generated by qRT-PCR detection using a 10-fold dilution gradient of the first cDNA (1, 10^-1^, 10^-2^, 10^-3^, 10^-4^) as templates with each repeated three times. Amplification efficiencies (E) and correlation coefficients (R^2^ values) were checked from standard curves. The qRT-PCR Ct values of eighteen genes were converted to relative copy numbers using the standard curves, and used to carry out geNorm and NormFinder and Bestkeeper analysis.

### 2.5. Validation of Reference Genes

To validate the reliability and accuracy of the selected reference genes for data normalization, the expression levels of seven genes were analyzed in different experimental conditions (Table S1). The expression data of the target genes were normalized with the two most stable genes and the least stable reference genes. The qRT-PCR amplification conditions were the same as those described above. The relative expression levels of all target genes were calculated by the 2^−∆∆Ct^ method.

### 2.6. Analysis of Candidate Reference Genes under Different Conditions Based on RNA-Seq Data

To analyze the expression stability of the eighteen CRGs, the RPKM (reads per kilobase per million mapped reads) values were gained from RNA-seq data in each experiment involved in different *L. edodes* strains, biotic/abiotic stresses and substrates. The RNA-seq data of 24 h *Trichoderma atroviride* stress in *L. edodes* strains YS3334 and YS55 and different carbon substrates for W1 were obtained from our lab studies (unpublished data). The expression data from heat (S606 and YS3357) and light (L135, ACCC 50903) treatments were obtained from previous studies [18,20]. The expression variation of the eighteen CRGs was determined using a threshold of an absolute log_2_ (fold change between treatment and control RPKM values) ≤ 1.

## 3. Results

### 3.1. Selection of Candidate Reference Genes and Primer Design

**Figure 1:**
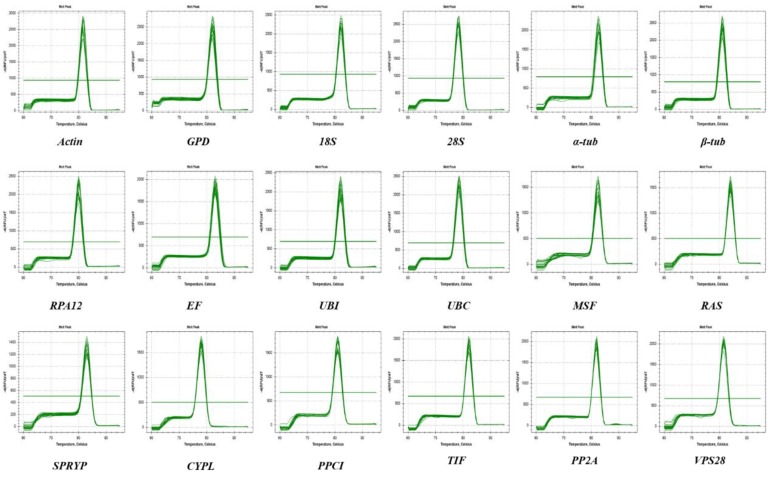
Amplification and dissolution curves of eighteen candidate reference genes.

Eighteen candidate genes, including seven house-keeping genes and eleven candidate genes, were selected according to previous reports in *Ganoderma lucidum*, *Volvaria volvacea*, *Pleurotus ostreatus*, *Morchella* sp. and *Lentinula edodes* [22,23,24,25,26,27]. The house-keeping genes were composed of actin (*Actin*), glyceraldehyde-3-phosphate dehydrogenase (*gpd*), 18S ribosomal RNA (*18S*), 28S ribosomal RNA (*28S*), β-tublin (*β-tub*), α-tublin (*α-tub*), and Elongation factor (*EF*). The eleven novel genes were implicated in DNA-directed RNA polymerase I subunit RPA12 (*RPA12*), Ubiquitin-protein ligase E3 (*UBI*), Ubiquitin-conjugating (*UBC*), MSF1-domain-containing protein (*MSF1*), Ras protein (*Ras*), SPRY-domain-containing protein (*SPRY*), Cyclophilin-like protein (*CYPL*), Peptidyl-prolyl cis-trans isomerase (*PPCI*), Translation initiation factor (*TIF*), Protein phosphatase 2A regulatory subunit B (*PP2A*) and Vacuolar protein sorting-associated protein 28 (*VPS28*). The information of the specific primers designed by Primer Premier 5.0 was displayed in Table 1. In addition, BLAST analysis revealed that the primers of every candidate reference gene were only matched to the target gene sequence in the *L. edodes* genome database. The 1% agarose gel electrophoresis result exhibited a specific single band for the PCR amplified products of the eighteen CRGs at an expected length (Figure S1), and the melting curve analysis also revealed a single peak for each PCR product (Figure 1). These results demonstrated a high specificity for all the primers used in this study.

### 3.2. Expression Profile of Candidate Reference Genes in L. edodes

**Figure 2:**
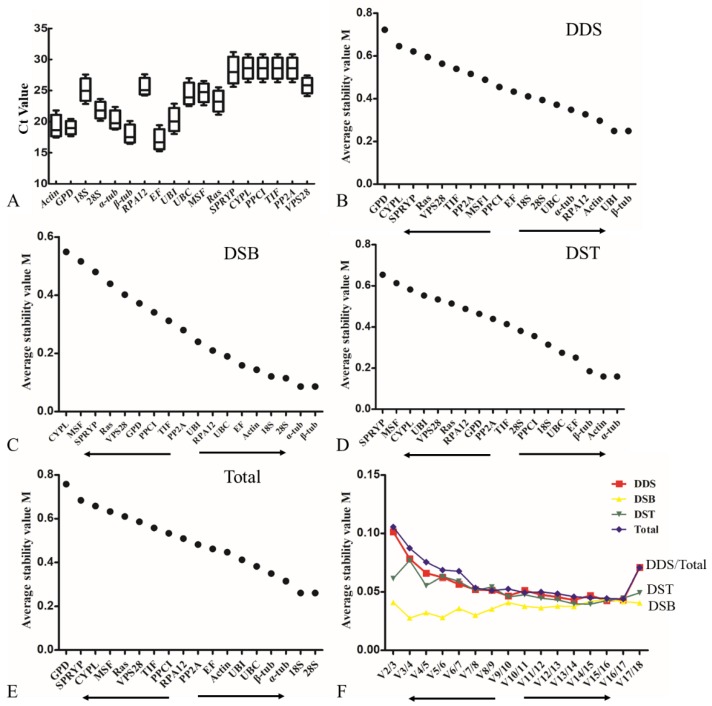
Expression stability analysis of eighteen candidate reference genes in *Lentinula edodes*. (**A**) variation in qRT-PCR values of eighteen candidate reference genes (CRGs) in all samples; (**B**–**E**) expression stability ranking of eighteen CRGs using geNorm under different development stages (DDS), different substrates (DSB), different stresses (DST) and all samples, respectively. (**F**) pairwise variation (Vn/Vn+1) showing the optional number reference genes required in an accurate normalization.

The cycle threshold (Ct) values were used to normalize for the standard curves generated with ten 10-fold serial cDNA dilutions by qRT-PCR. The formula E = 10 ^-1/slope^ -1 was used to calculate the amplification efficiency (E) varied from 90.6% for *28S* to 102.3% for *MSF1* (Table 1). All correlation coefficients (R^2^) of eighteen genes were greater than 0.99, indicating a reliable linear relationship of the respective Ct values with the log values of the initial gene copy numbers. The mean Ct values of the eighteen candidate genes in all samples under different conditions varied from 15.25 to 30.84 (Figure 2A), and ten of the candidate genes had a Ct mean values lower than 25, including *Actin*, *GP*D, *α-tub*, *β-tub*, *EF*, *28S* and *UBI*, demonstrating that the expression levels of they were very high in all samples. *EF* showed considerably higher expression than the other candidate reference genes (low Ct values), while *SPRYP* exhibited the lowest expression (highest Ct values) in all samples. Relative variation analysis of the Ct values (the maximum Ct value minus the minimum Ct value) in all candidate reference genes documented the greatest variation in *SPRYP* while the smallest variation in *GPD* of *L. edodes*.

### 3.3. Evaluation of Expression Stability of the Eighteen Candidate Reference Genes in L. edodes

According to qRT-PCR Ct values, three statistical algorithms geNorm and NormFinder and BestKeeper were used to evaluate the stability of the eighteen selected CRGs. All selected genes were used for geNorm and NormFinder analyses, while, for BestKeeper analysis, only the top ten stable genes obtained with the two previous algorithms were used.

GeNorm analysis of candidate reference genes. The expression stability measure (M) of a CRG was calculated as previously reported [36]. The smaller the M values are, the higher the expression stability will be. The values of the eighteen CRGs varied from 0.758 to 0.086 (Figure 2 and Table S2). From the perspective of different development stages, *β-tub* and *UBI* were the most stable genes with the smallest value of 0.249, followed by *Actin* and *RPA12*, in contrast to the least stable genes of *GPD* and *CYPL* with M values of 0.723 and 0.646, respectively (Figure 2B). Under the conditions of different substrates, *α-tub* and *β-tub* were most stable with the smallest value of 0.086, while *CYPL* and *MSF* were less stably expressed (Figure 2C). In terms of different stresses, *α-tub* and *Actin* were the most stably expressed with an M value of 0.159, whereas *SPRYP* and *MSF* were less stably expressed (Figure 2D). All the integrated data suggested that *28S* and *18S* were the most stably expressed genes, while *GPD* and *SPRYP* were the least stably expressed genes (Figure 2E). In addition, the pairwise variation values (Vn/Vn+1) calculated by geNorm were used to determine the optional number of reference genes for accurate normalization. As reported previously, an additional reference gene would be unnecessary with the pairwise variation value less than 0.15 [37]. As shown in Figure 2F, all pairwise variation values were smaller than 0.15, demonstrating that two genes are sufficient for accurate normalization under different conditions of *L. edodes*. The optimal combination of reference genes was *UBI* with *β-tub* for different development stages, *β-tub* with *α-tub* for different substrates and *α-tub* with *Actin* for different stresses, respectively. Nevertheless, *28S* with *18S* was the optimal combination of reference genes under all conditions (Table S2). 

NormFinder analysis of candidate reference genes. The CRGs were ranked by NormFinder based on the intra and inter group expression variations [36]. A notable difference was observed between the results of NormFinder and geNorm analyses about the most and least stably expressed genes. In NormFinder analysis, *UBI* and *RPA12* were predicated as the most stable genes for different substrates and different development stages, respectively, while *α-tub* was defined as the most stable gene under different stress and total conditions (Table 2). Meanwhile, ranked in the second and third positions were *RPA12* and *18S* for different substrates, *α-tub* and *Actin* for different development stage, *18S* and *PPCI* for different stresses, and *28S* with *β-tub* for total conditions. The least stable genes were *CYPL* for different substrates, *SPRYP* for different stresses and *GPD* for different development stages and total conditions.

BestKeeper analysis of candidate reference genes. The top ten CRGs ranked by the sum of stability values in geNorm and NormFinder analyses were selected for BestKeeper analysis. The expression variability of the top ten CRGs was measured by the standard deviation (SD) and correlation coefficient (R). The lower the values of SD and CV were, the higher the stability of CRGs would be. The CRGs with a SD value larger than 1 were considered as unstably expressed genes [38]. As shown in Table 3, *RPA12* and *28S* were defined as the most stable genes for different substrates, while *18S*, *PPCI* and *UBC* with a low R value were identified as unstably expressed genes. In terms of different development stages, the most stable gene was *Actin*, followed by *RPA12* and *UBC* ranked in the second and third positions, respectively. Meanwhile, *28S* was identified as the most stably expressed gene under different stresses, followed by *PPCI* and *RPA12* in the second and third positions, respectively. Nevertheless, *18S* was eliminated due to its high *p*-value (0.08866) and low R value (0.6380). The CRGs with the low *p*-values (0.001) and a high correlation coefficient (>0.93) were defined as the most stably expressed genes, including four genes (*RPA12*, *PPCI*, *28S* and *Actin*).

Based on the results of the three statistical analyses, *Actin* and *RPA12* were defined as the two most stable genes in different stages, while *28S* with *RPA12* and *α-tub* with *Actin* were defined as the most stably expressed for different substrates and different stresses, respectively. In summary, *28S* could be treated as the reference gene under all different conditions.

### 3.4. Validation of Selected Reference Genes in L. edodes

To validate the reliability of the optimal and least reference genes for qRT-PCR analysis under different conditions, the relative expression levels of seven genes were calculated. 

**Figure 3:**
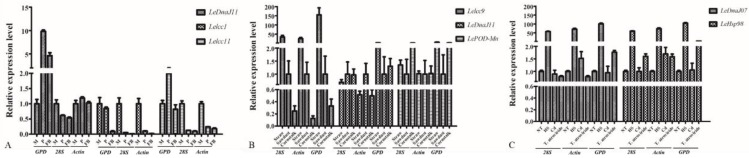
The relative expression levels of seven genes normalizing by the selected reference genes under different experimental conditions. (**A**) qRT-PCR analysis of three genes normalizing by the selected reference genes during different development stages; (**B**) qRT-PCR analysis of three genes normalizing by the selected reference genes in different substrates; (**C**) qRT-PCR analysis of three genes normalizing by the selected reference genes under different stress conditions.

From the perspectives of growth and development stages, it was documented that the relative expression levels of three tested genes exhibited a significantly difference using different reference genes (Figure 3A). In comparison to the mycelia stage, *LeDnaJ11* normalized by *GPD* was obviously upregulated under primordium and fruiting body stage, whereas there was no difference found in the analysis with *Actin* and *28S* as the reference genes. Meanwhile, we found that *Lelcc1* and *Lelcc11* in primordium stage were significantly downregulated when normalized by *Actin* and *28S*, but no obvious difference was detected with the reference gene *GPD*.

In terms of different substrates, an obvious difference was found in qRT-PCR analysis normalized by different reference genes (Figure 3B). The relative expression level of *Lelcc9* in straw medium exhibited a similar upregulation trend using different reference genes, but the upregulation range with *GPD* was significantly higher than those of *Actin* and *28S*. In addition, it was observed that the relative expression levels of *LeDnaJ11* and *LePOD-Mn* normalized by *GPD* showed an obvious upregulation in straw medium, whereas no difference was found in the analysis with *Actin* and *28S* as the reference genes.

Under different stress conditions, a prominent difference was detected in qRT-PCR analysis normalized by different reference genes (Figure 3C). It was found that the relative expression levels of *LeDna98* normalized by *GPD* showed an obvious upregulation when subjected to *T. atroviride* infection, yet no difference was observed in the analysis with *Actin* and *28S* as the reference genes. In addition, the relative expression levels of *LeDnaJ07* and *LeHsp98* in heat stress showed a similar change trend using different reference genes, where the upregulation range with *GPD* was higher than those of *Actin* and *28S*.

### 3.5. Evaluation of the Expression Stability of Candidate Reference Genes in Different L. edodes Strains

**Figure 4:**
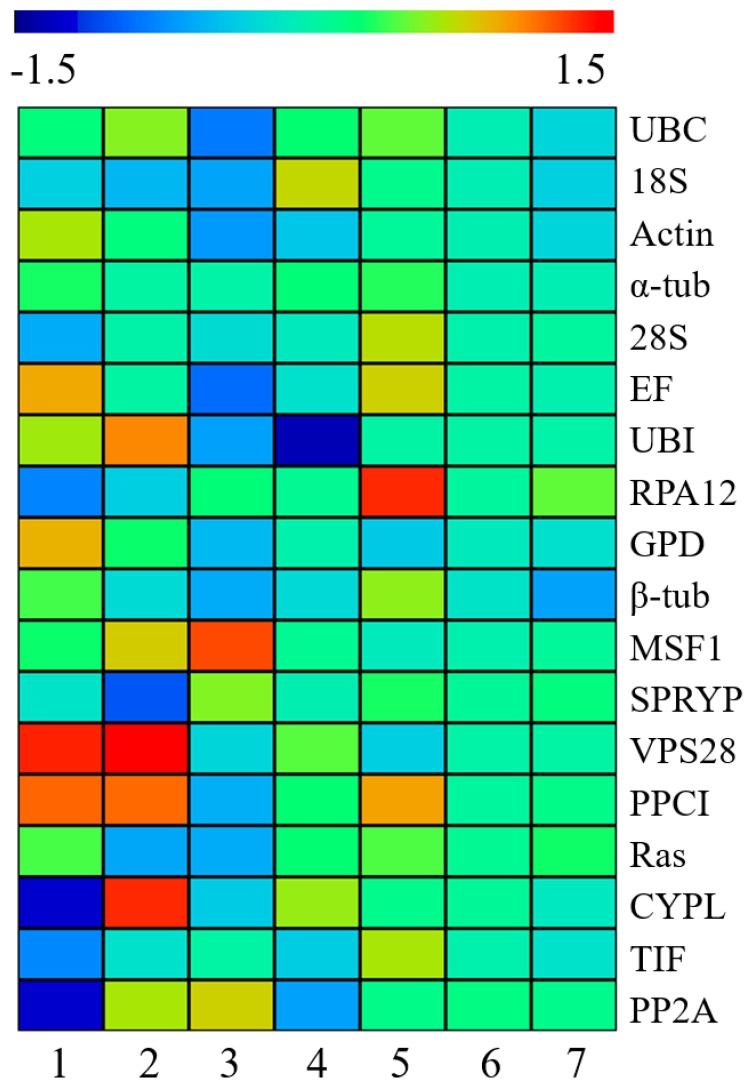
Heat map (log_2_fold change) showing expression stability of eighteen candidate reference genes in different *L. edodes* strains under different conditions. (**1** and **2**) heat stress/control in YS3357 and S606; (**3** and **4**) *Trichoderma atroviride* stress/control in YS3334 and YS55; (**5** and **6**) cellulose/glucose and cellulose+ sodium lignosulphonate/glucose in WX-1. 7, Light treatment/control in L135. Red, Upregulation. Blue, Downregulation. Scale bar is given at the top of the heat map.

To estimate the stability of the selected CRGs in different *L. edodes* strains, the RPKM values were gained from RNA-seq databases [20,37]. Genes with the RPKM ratio between any two treatments displaying a less than 2-fold change between treatment and control (non-treated) samples were considered as stably expressed. Under heat stress, for *L. edodes* strain YS3357, *VPS28* and *PPCI* displayed an obvious upregulation, in contrast to the downregulation of *CYPL* and *PP2A*; for *L. edodes* strain S606, four genes (*UBI*, *PPCI*, *VPS28* and *CYPL*) were upregulated, in contrast to the downregulation of *SPRYP* (Figure 4 and Table S3). From the perspective of *T. atroviride* infection, sixteen of eighteen CRGs were stably expressed except for *UBI* (downregulated in YS55) and *MSF* (upregulated in YS3334). During the coloring process in the presence or absence of light, only one gene *RPA12* showed upregulation in *L. edodes* strain L135. When the mycelia of *L. edodes* strain W-1 mycelia were cultured in the CYM liquid medium containing different carbon sources (glucose, cellulose or cellulose and sodium lignosulphonate), all of the CRGs were stably expressed (Figure 4 and Table S3). 

## 4. Discussion

Abiotic and biotic stresses and cultivation materials are the major limiting factors for the yield and quality traits of *L. edodes*. To solve these problems, we need to understand the molecular mechanism of *L. edodes* in response to stresses and the breeding of stress-resistant varieties. qRT-PCR can help us to understand this mechanism by quantifying the gene expression, and a stable internal gene reference gene is a prerequisite to ensure the reality of qRT-PCR results. Recently, an increasing number of studies have been performed on reference genes in organisms. For instance, several house-keeping genes were found to be unstably expressed under abiotic and biotic stresses [39,40], suggesting the necessity to identify reliable reference genes for qRT-PCR analysis. From the perspective of macro basidiomycetes, the stabilities of novel and traditional candidate genes have been estimated undertaken in *G. lucidum*, *V. volvacea*, *P. ostreatus* and *L. edodes* under different development stages, strains, nutrient conditions and abiotic stresses [22,23,24,25,26,27,40]. These reports suggested that the use of reference genes should be based on specific stress conditions and sample tissues as well as strains.

In the present study, a comprehensive analysis of eighteen CRGs, including traditional and novel reference genes from previous studies, was performed under different stresses (40 °C heat, Cd^2+^ excess and *T. atroviride* infection), different substrates (straw, sawdust and corn stalk) and different development stages (mycelia, primordia and fruit bodies). The melting curves as well as R^2^ and E values exhibited high specificity and amplification efficiency for all eighteen CRGs (Figure 1 and Table 1). 

Under heat and Cd^2+^ as well as *T. atroviride* stress conditions, *α-tub* was demonstrated as the best reference gene by the results from the three statistical analyses, which was consistent with previous studies reporting that *α-tub* was stably expressed under many kinds of abiotic stresses in fungi (*V. volvacea* and *L. edodes*), animals and plants [23,26,41,42,43]. *PPCI* and *Actin* were identified as the second most stable genes by the three statistical methods. Previous reports documented *Actin* as a stably expressed gene in the response of *L. edodes* to 37 °C stress and in tobacco and Jute subjected to abiotic stresses [26,44,45] and *CYC3* (*PPCI*) as a reliable reference gene for *Morchella* sp. in temperature stress [24]. The pairwise variation V2/3 value was below 0.15, demonstrating that the two genes were suitable for normalization in gene expression analysis. Therefore, *α-tub* with *Actin* or *PPCI* was suggested as the pair of reference genes for *L. edodes* in response to different kinds of stresses. 

In different substrates, *28S* and *RPA12* were determined by the three analysis methods as the most suitable primer pair of reference genes. The analysis of geNorm, NormFinder and BestKeeper ranked *28S* in the fourth, third and second position, respectively. *RPA12* was estimated as the most stable gene by BestKeeper analysis and ranked in the second position by geNorm analysis. For the entomopathogenic fungus *Beauveria bassiana*, *28S* was confirmed as the most stably expressed gene under a number of nutritional and stress conditions [46]. However, *RPA12* encoding DNA-directed RNA polymerase I subunit has rarely been reported as an optimal reference gene in previous studies. Therefore, *28S* was recommended as the best internal control gene for the response of *L. edodes* to different substrates.

For different development stages, the selection of reference genes has been reported in different varieties of edible fungi. In *V. volvacea*, *Ras* and *SPRYP* were reported as the most stable genes in heterokaryon H1521 [40], while *L-asp* and *MSF* were considered as stably expressed genes in homokaryon PYd15 [23], which were different from the present study in that *Ras* and *SPRYP* as well as *MSF* were found to be unstably expressed in all samples. Additionally, *CYC3* encoding peptidyl-prolyl cis-trans isomerase exhibited a stable expression in ten *Morchella* species [24]. In *L. edodes* strain Xin 808, *18S* and *Rpl4* (ribosomal protein L4) were confirmed as the stably expressed genes [27], while in, *L. edodes* strain, W-1 *Actin* and *RPA12* were defined as the stably expressed genes in different development stages. Furthermore, *Actin* was suggested as the most relevant reference genes during berry development [47], and *ACT1* was reported as the most suitable reference gene during rice seed development [48]. All these results indicate that *Actin* can be used as a reliable reference gene during *L. edodes* development.

## 5. Conclusions

Overall, the three different statistical algorithms identified α-tub as the optimal internal control gene for many kinds of stresses and different *L. edodes* strains, while Actin and 28S as the suitable reference genes for gene expression normalization in different development stages and substrates. This study contributes to accurate analysis of differential expression changes in *L. edodes* under different conditions.

## Figures and Tables

**Table 1:** Delineations of eighteen candidate candidate reference genes in *L. edodes* and parameters gained by qRT-PCR analysis.

Symbol	Gene ID	Gene Name	Forward Primer Sequence	Reverse Primer Sequence	Size (bp)	Efficiency (%)	R^2^
Actin	LE01Gene01050	Actin	GCATCCTGTCCTTCTTACCGAG	AAGAGCGAAACCCTCGTAGATG	214	96.1	0.998
GPD	LE01Gene07114	Glyceraldehyde-3-phosphate dehydrogenase	CCTTCCGCTGATGCACCTAT	GTTGACAGAACGACCGCCAC	240	94.1	0.998
18S	LE01Gene00881	18S ribosomal RNA	ATGCTGGCTCCGTTCGC	AGGTGCTCCCCGCTTCTTA	168	97.5	0.988
28S	LE01Gene02296	28S ribosomal RNA	GCCTGCCACAAAGGATGAAA	GCTCCAAGCCTAAGAACAGTCCA	157	90.6	0.992
β-tub	LE01Gene08622	β-tubulin	CAGACCCAAGACACGGACG	TGGCAGTAGAGTTACCCAGGAA	213	93.2	0.998
α-tub	LE01Gene01435	α-tubulin	TCCAACTTGAACAGGCTTATCG	AACGGAAAGTGAATACGAGGGA	134	94.9	0.994
RPA12	LE01Gene06896	DNA-directed RNA polymerase I subunit RPA12	TCCGAAAGATAGCGAACCGAA	GGAGACCAAATCGCCCAAGT	184	97.2	0.998
EF	LE01Gene03252	Elongation factor	ACTTCCCAGGCTGATTGTGCT	TCGCTCCATTTGGTGGTGTC	170	96.6	1
UBI	LE01Gene06776	UBI Ubiquitin-protein ligase E3	ATCACGGTCACGAAAGAACAACT	CTTACATTCCAAAACTCGCACAGA	171	96	0.998
UbC	LE01Gene00040	Ubiquitin-conjugating	GGCGGTCCAGTTTGTTGTCA	CGGTCGGTGTTTCTCCTTGC	178	95.6	0.993
MSF1	LE01Gene08077	MSF1-domain-containing protein	TATTCGCCTTCGTCAACACCT	ACCGCTGAGCCATCCACCT	118	102.3	0.997
Ras	LE01Gene13143	Ras protein	AGGTCGGGATGAATGAGGG	CTTCGTCGTTTGGATCTTTGC	222	99.5	0.999
SPRYP	LE01Gene00010	SPRY-domain-containing protein	ATGTCAAACTGTCCCGTCTTCC	CCATAAGGTGTTCCGTTTCGTT	103	91.2	0.998
CYPL	LE01Gene07769	Cyclophilin-like protein	AGTGGTGTACTCCCTGATTTTGTC	GGTCTGCGTCGCCCTTTT	100	92.9	0.997
PPCI	LE01Gene06576	Peptidyl-prolyl cis-trans isomerase	AGGACGAATTGCATCCAGAAC	GGGTAGGACCAAGAGTCAAGAAG	111	97.6	0.997
TIF	LE01Gene09672	Translation initiation factor	ACCGCCGTAAAACGAGTAGC	CCTGGTTGCGAGGTGAATG	203	101.5	0.998
PP2A	LE01Gene09754	Protein phosphatase 2A regulatory subunit B	TCGGCTGACGATTTGCG	GAGTAGTGGGGTCTTCCTCTTCTT	253	100.9	0.998
VPS28	LE01Gene10038	Vacuolar protein sorting-associated protein 28	CTTCAAGGGCAGCAAGGATT	TGGCGTGACTGTTCTTCGGTA	108	100.9	0.999

**Table 2:** Stability analysis of eighteen candidate reference genes by NormFinder.

Rank	Different Substrates		Different Development Stages		Different Stresses		Total	
	NormFinder	Stability Value	NormFinder	Stability Value	NormFinder	Stability Value	NormFinder	Stability Value
1	UBI	0.077	RPA12	0.042	α-tub	0.124	α-tub	0.161
2	RPA12	0.09	α-tub	0.15	18S	0.172	28S	0.204
3	18S	0.152	Actin	0.186	PPCI	0.184	β-tub	0.231
4	28S	0.165	β-tub	0.188	28S	0.21	18S	0.242
5	EF	0.172	UBI	0.198	TIF	0.23	PP2A	0.292
6	UBC	0.19	PPCI	0.254	Actin	0.234	RPA12	0.303
7	TIF	0.209	28S	0.314	β-tub	0.246	PPCI	0.306
8	Actin	0.228	MSF	0.314	PP2A	0.284	Actin	0.309
9	PPCI	0.235	UBC	0.318	GPD	0.317	UBI	0.314
10	α-tub	0.238	EF	0.319	UBC	0.321	UBC	0.316
11	β-tub	0.248	PP2A	0.339	Ras	0.332	EF	0.352
12	PP2A	0.26	18S	0.351	EF	0.353	TIF	0.357
13	VPS28	0.329	VPS28	0.395	VPS28	0.368	VPS28	0.38
14	GPD	0.355	TIF	0.424	RPA12	0.38	Ras	0.431
15	Ras	0.401	SPRYP	0.46	UBI	0.399	MSF	0.445
16	SPRYP	0.443	CYPL	0.501	CYPL	0.452	CYPL	0.488
17	MSF	0.478	Ras	0.504	MSF	0.521	SPRYP	0.536
18	CYPL	0.502	GPD	0.88	SPRYP	0.61	GPD	0.879

**Table 3:** Stability analysis of eighteen candidate reference genes by BestKeeper.

	Actin	18S	28S	RPA12	UBC	UBI	EF	α-tub	β-tub	PPCI
Different substrates										
std dev [± Ct]	0.54	0.35	0.42	0.39	0.31	0.48	0.52	0.52	0.57	0.39
coeff. of corr. [R]	0.998	0.485	0.965	0.995	0.897	1	0.977	0.964	0.997	0.757
Ranking	6	10	2	1	8	3	4	5	7	9
Different development stages										
std dev [± Ct]	0.34	0.92	0.87	0.42	0.44	0.72	0.44	0.68	0.64	0.57
coeff. of corr. [R]	0.994	0.979	0.995	0.924	0.947	0.971	0.806	0.96	0.994	0.889
Ranking	1	8	7	2	3	6	10	5	4	9
Difference stresses										
std dev [± Ct]	1.27	0.86	0.84	0.95	1.34	1.33	1.39	1.17	1.28	0.93
coeff. of corr. [R]	0.999	0.283	0.998	0.928	0.964	0.969	0.986	1	0.992	0.993
Ranking	5	10	1	3	8	7	9	4	6	2
Total samples										
std dev [± Ct]	1.00	0.94	0.98	0.78	1.03	1.19	1.02	1.11	1.14	0.85
coeff. of corr. [R]	0.957	0.638	0.967	0.943	0.973	0.977	0.954	0.991	0.991	0.937
*p*-value	0.001	0.089	0.001	0.001	0.001	0.001	0.001	0.001	0.001	0.001
Ranking	4	10	3	1	6	9	5	7	8	2

## Supplementary Materials

The following are available online at https://www.mdpi.com/2073-4425/10/9/647/s1, Figure S1: Amplification fragments of eighteen candidate reference genes by agarose gel electrophoresis, Table S1: Delineations of genes used for validation analysis of selected reference genes, TableS2: Stability analysis of eighteen CRGs was calculated by geNorm, TableS3: Transcriptomic analysis of eighteen CRGs in *L. edodes* strains under different conditions.
