# Supplementary material for: Selection and Validation of Reference Genes for qRT-PCR in Lentinula edodes under Different Experimental Conditions
Source: Genes (Basel). 2019 Aug 27;10(9):647. doi: 10.3390/genes10090647 (PMC6770232; doi:10.3390/genes10090647)

**Figure S1.** Amplification fragments of eighteen candidate reference genes by agarose gel electrophoresis.


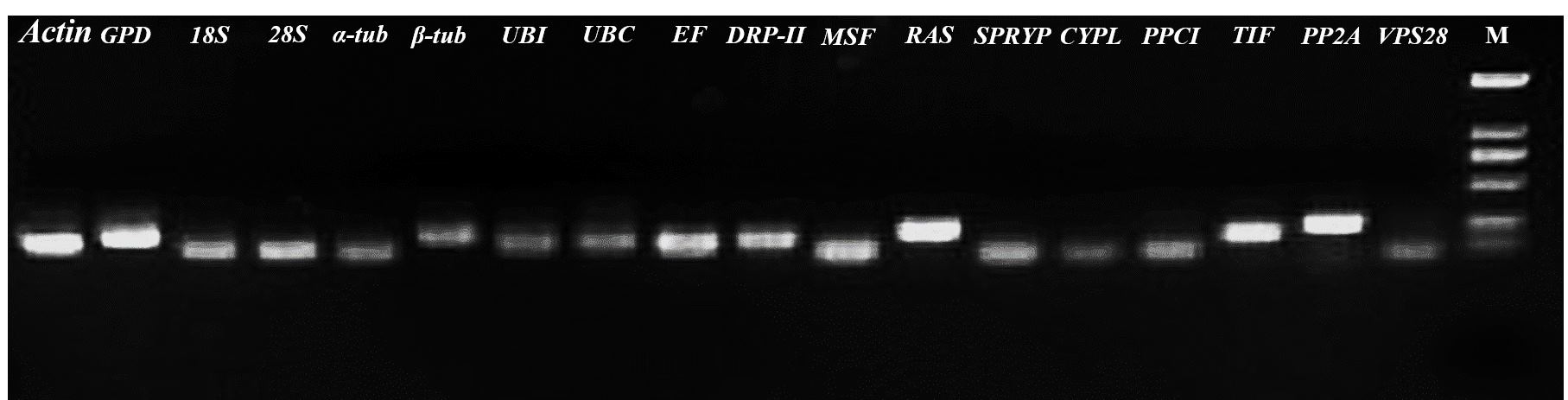

Supplement: Supplementary file 1 [file genes-10-00647-s001.zip › genes-564185-SI/genes-564185 supplimentary figureS1.docx]
